# *MaMHR* Contributes to Conidiation, Stress Adaptation, and Virulence in *Metarhizium acridum*

**DOI:** 10.3390/jof12100718

**Published:** 2026-09-24

**Authors:** Guilin Li, Ke Li, Yuxian Xia

**Affiliations:** 1 Genetic Engineering Research Center, School of Life Sciences, Chongqing University, Chongqing 401331, China; 2 Chongqing Engineering Research Center for Fungal Insecticide, Chongqing 401331, China; 3 Key Laboratory of Gene Function and Regulation Technologies Under Chongqing Municipal Education Commission, Chongqing 401331, China; 4 National Engineering Research Center of Microbial Pesticides, Chongqing 401331, China

**Keywords:** entomopathogenic fungus, reactive oxygen species, nucleotide excision repair, melanin biosynthesis, *Locusta migratoria*, stress tolerance

## Abstract

*Metarhizium acridum* is an important entomopathogenic fungus widely used for biological control of locust pests. Although Zn_2_Cys_6_ transcription factors regulate diverse fungal developmental and stress-responsive processes, the functions of MHR-containing Zn_2_Cys_6_ transcription factors remain poorly understood, particularly in entomopathogenic fungi. Here, we characterized *MaMHR*, a previously uncharacterized Zn_2_Cys_6_ transcription factor containing a fungal transcription factor middle homology region (MHR), through targeted gene deletion and complementation in *M. acridum*. Loss of *MaMHR* delayed conidial germination, reduced conidial production by approximately 17.6% and 28.3% on 1/4 SDAY and SYA media, respectively, and shifted conidiation from the microcycle to the normal mode. The Δ*MaMHR* mutant also showed impaired tolerance to UV-B irradiation and wet heat, with UV-B and heat IT_50_ values reduced by approximately 26.5% and 22.6%, respectively, as well as reduced tolerance to selected chemical stresses. Furthermore, *MaMHR* deletion reduced melanin accumulation by approximately 47.0%, disturbed reactive oxygen species (ROS) homeostasis, altered antioxidant enzyme activities, and affected the expression of nucleotide excision repair-related genes following UV-B stress, whereas trehalose accumulation was unaffected. Disruption of *MaMHR* also reduced fungal virulence during topical infection as reflected by an approximately 14.6% increase in LT_50_, and reduced cuticle penetration-associated fungal outgrowth, whereas virulence following intra-hemocoel injection was not significantly affected. Together, these findings demonstrate that *MaMHR* contributes to fungal development, environmental stress adaptation, and host infection, potentially through regulation of oxidative stress, melanin biosynthesis, and DNA repair-associated responses.

## 1. Introduction

Entomopathogenic fungi are important biological control agents for sustainable pest management due to their environmental safety, host specificity, and low risk of resistance development. Among them, *Metarhizium acridum* is a highly host-specific fungal pathogen of locusts and has been widely applied in biological control programs targeting locust outbreaks [1].

Transcription factors play central roles in regulating fungal growth, development, metabolism, and environmental adaptation [2,3]. Among these, Zn_2_Cys_6_-type transcription factors represent one of the largest classes of fungus-specific regulatory proteins and are involved in diverse biological processes [4,5], including carbon and nitrogen metabolism, secondary metabolism, conidiation, development, and stress responses [6,7]. However, the functions of a subset of Zn_2_Cys_6_ transcription factors remain poorly understood [8].

The fungal transcription factor middle homology region (MHR) is conserved among diverse fungal species and is present in a subset of Zn_2_Cys_6_-type transcription factors [9,10,11]. In *Candida albicans*, the Zn_2_Cys_6_ transcription factor Tac1 contains an MHR domain that is involved in xenobiotic sensing and activates drug efflux pump genes, thereby contributing to antifungal resistance [12]. In *Myceliophthora thermophila*, the MHR-containing transcription factor MHR1 was shown to regulate cellulase and xylanase production [10]. These findings suggest that MHR domain-containing transcription factors play important roles in fungal metabolic regulation and environmental adaptation [10,12,13].

However, functional characterization of MHR domain-containing proteins in entomopathogenic fungi remains limited. In *Metarhizium acridum*, the C_2_H_2_ zinc finger protein MaNCP1 has been shown to regulate conidiation through the nitrate assimilation pathway [14], highlighting the importance of transcriptional regulation in fungal development [14,15]. Nevertheless, whether MHR-containing Zn_2_Cys_6_ transcription factors are involved in conidiation, stress responses, and pathogenicity in *M. acridum* has not been systematically investigated [16].

In this study, we identified a Zn_2_Cys_6_ transcription factor containing both a GAL4-like DNA-binding domain and an MHR domain in the genome of *M. acridum*, which was designated *MaMHR*. Phylogenetic analysis and protein domain analysis were performed to characterize its evolutionary and structural features. Gene deletion and complementation mutants were constructed to investigate its biological functions.

Phenotypic analyses were conducted to evaluate its roles in fungal development [17], including conidiation pattern, conidial germination, and conidial production, as well as in environmental stress responses, including UV-B irradiation, heat shock, and chemical stress. Furthermore, physiological and biochemical assays were performed to explore the underlying regulatory mechanisms. In this study, we aimed to determine whether *MaMHR* contributes to conidiation, stress tolerance, and virulence through effects on oxidative stress homeostasis, melanin biosynthesis, and DNA damage responses, thereby providing new insights into the regulatory networks governing development and environmental adaptation in *M. acridum*. *M. acridum* is highly specific to acridid grasshoppers and locusts, including *Locusta migratoria* and *Schistocerca gregaria*, and has been developed and commercialized as a mycoinsecticide for locust control in several countries. Its narrow host range and favorable environmental safety profile make it an important entomopathogen for the biological control of locusts and grasshoppers and an attractive alternative to conventional chemical insecticides [18].

## 2. Materials and Methods

### 2.1. Fungal Strains and Culture Conditions

The experimental methods used in this study were adapted from previously established protocols with minor modifications where indicated. Conidial germination, conidiation pattern observation, stress tolerance assays, melanin extraction, and virulence bioassays were performed following procedures described previously [5,14,19,20].

The wild-type *Metarhizium acridum* strain CQMa102 (CGMCC No. 0877) was routinely cultured on 1/4 strength Sabouraud dextrose agar with yeast extract (1/4 SDAY; containing 1% glucose, 0.25% peptone, 0.5% yeast extract, and 1.8% agar; pH 6.4–6.5). SYA medium contained (per liter): sucrose 30 g, yeast extract 5 g, NaNO_3_ 3 g, MgSO_4_ 5 g, KCl 5 g, KH_2_PO_4_ 1 g, FeSO_4_ 0.01 g, MnSO_4_ 0.01 g, and agar 18 g, with the pH adjusted to 6.4–6.5 [14]. *Escherichia coli* competent cells DH5α and *Agrobacterium tumefaciens* strain AGL were purchased from Coolaber Technology Co., Ltd. (Beijing, China) and Weidi Biotechnology Co., Ltd. (Shanghai, China), respectively. The gene knockout vector pK2-PB and the complementation vector pK2-eGFP-Sur were kindly provided by the Gene Engineering Center of Chongqing University (Chongqing, China).

### 2.2. Bioinformatics Analysis

The nucleotide (NW_027094046.1) and protein sequences (XP_007812188.1) of *MaMHR* and its homologs were retrieved from the National Center for Biotechnology Information (NCBI) database (https://www.ncbi.nlm.nih.gov/ (accessed on 1 July 2026)). The ProtParam tool on the ExPASy server (https://web.expasy.org/protparam/ (accessed on 1 July 2026)) was used to predict the molecular weight and theoretical isoelectric point (pI) of the protein. Conserved domain analysis was performed using the NCBI Conserved Domain Database (CDD). Multiple sequence alignment was conducted using DNAMAN v7.1. A phylogenetic tree was constructed using the neighbor-joining method with 1000 bootstrap replicates and the p-distance model in MEGA v7.0 with default parameters.

### 2.3. Gene Knockout and Complementation

Based on bioinformatics analysis of *MaMHR*, the upstream and downstream homologous arms of the *MaMHR* gene were respectively amplified and ligated into the pK2-PB vector containing the phosphinothricin resistance gene to construct the knockout construct. For complementation, the coding sequence of the target gene, together with its ~2-kb upstream promoter region and downstream terminator fragment, was amplified and inserted into the pK2-eGFP-Sur vector carrying the chlorimuron ethyl resistance gene to generate the complementation vector. These constructs were introduced into *Metarhizium acridum* via *Agrobacterium tumefaciens*-mediated transformation. Primary transformants were selected on Czapek–Dox agar supplemented with 400 μg/mL phosphinothricin for knockout, or 100 μg/mL chlorimuron ethyl for complementation [14]. Putative knockout and complementation mutants were confirmed by junction PCR with locus-specific primers and subsequent Sanger sequencing, and transcriptional restoration was verified by RT-qPCR. All primers used for vector construction, mutant verification, and RT-qPCR are listed in Table S1, and the knockout/complementation strategy and validation results are shown in Figure S1.

### 2.4. Determination of Germination Rate, Sporulation Yield, and Observation of Conidiation Patterns

A 100-µL aliquot of spore suspension was spread onto 1/4 SDAY medium and incubated inverted at 28 °C. At 2-h intervals, agar blocks were excised and examined under a microscope to observe spore germination. For each strain, three independent biological replicates were analyzed, with 300 spores counted per replicate to calculate the germination rate and GT_50_ was calculated as the time required for 50% of conidia to germinate under non-stress conditions. The germination rate was calculated as: (number of germinated spores/total number of spores) × 100%. A conidium was considered germinated when the germ tube length was equal to or longer than half of the conidial diameter.

To assess sporulation yield, 2-µL droplets of spore suspension were spotted onto 1/4 SDAY and SYA plates (six replicates for each medium). At 3, 6, 9, 12, and 15 days post-inoculation, the mycelial mats were harvested and ground. The resulting suspensions were diluted appropriately, and the conidia were counted using a hemocytometer.

For observation of conidiation patterns, mature conidia of the wild-type, Δ*MaMHR*, and complementation (CP) strains were collected and suspended in 0.05% Tween-80 to a final concentration of 1 × 10^7^ conidia/mL. A 100-µL aliquot of each suspension was spread onto 1/4 SDAY and SYA plates. Starting at 12 h post-inoculation, the plates were examined microscopically every 4 h until 36 h to record the conidiation mode. The determination of conidial germination and conidiation patterns was performed following previously described procedures [14,19].

### 2.5. Analysis of Stress Tolerance

#### 2.5.1. Physical Stress

Stress tolerance assays were performed following previously described procedures [5]. Spore suspensions of the wild-type, Δ*MaMHR*, and complementation (CP) strains were aseptically prepared to a final concentration of 1 × 10^7^ spores/mL. Aliquots of 100 µL of each suspension were spread onto 1/4 SDAY plates. The plates were then exposed to UV-B radiation (1350 mW/m^2^ for 0.5–2.5 h) or subjected to a 45 °C water-bath treatment (for 2–12 h). Following these treatments, the plates were incubated inverted at 28 °C for 20 h, and spore germination was examined under a microscope. The 50% inhibition time (IT_50_), defined as the exposure time causing 50% germination inhibition, was estimated by fitting the germination rates at different exposure times to a four-parameter logistic model using nonlinear regression in GraphPad Prism 9.5.0, and the 95% confidence intervals were calculated. Statistical comparisons of IT_50_ values among strains were performed using one-way ANOVA followed by Tukey’s post-hoc test. For each treatment, 300 conidia were counted, and the germination rate was calculated as: (number of germinated spores/total number of spores) × 100%.

#### 2.5.2. Chemical Stress

Chemical stress assays were performed as described previously [5,14]. Separately, spore suspensions were adjusted to 1 × 10^6^ spores/mL. Two-microliter droplets of each suspension were spotted onto 1/4 SDAY plates supplemented with 182 g/L sorbitol (SOR), 58 g/L NaCl, 0.6 mL/L 30% H_2_O_2_, 0.01% SDS, 0.5 mg/mL Congo Red (CR), or 0.05 mg/mL Calcofluor White (CFW). The plates were incubated at 28 °C for 6 days, after which the colony diameters were measured, photographs were taken, and the relative growth rate was calculated based on the measured colony sizes. Relative growth rate = (colony diameter on stressed medium/colony diameter on control medium) × 100%.

### 2.6. Investigation of Stress Resistance Mechanisms

#### 2.6.1. Determination of Melanin Content

Spore suspensions of the wild-type (WT), Δ*MaMHR*, and complementation (CP) strains were aseptically prepared to a final concentration of 1 × 10^8^ spores/mL. A 1-mL aliquot of 1 M NaOH was added to the spore suspension, and the mixture was heated in a boiling water bath for 2 h. After centrifugation, the supernatant was collected for use. Melanin standards (Sigma-Aldrich, synthetic melanin) were diluted with distilled water to concentrations of 0, 10, 20, 40, 80, 120, and 160 µg/mL. The absorbance (A) of each sample was measured at a wavelength of 400 nm. In parallel, the expression levels of key melanin biosynthesis genes (*PksP*, *SCD*, *THR*, *Abr1*, *Tyr*, *HppD*, and *LAC*) were examined by RT-qPCR. Melanin biosynthesis-related gene expression was analyzed in conidia collected from 15-day-old cultures grown on 1/4 SDAY medium under standard (non-stress) conditions [20].

#### 2.6.2. Assessment of Nuclear Morphology and DNA Repair-Related Gene Expression

Spore suspensions of the WT, Δ*MaMHR*, and CP strains were aseptically prepared to a final concentration of 5 × 10^7^ spores/mL. A 100-µL aliquot of each suspension was evenly spread onto 1/4 SDAY plates. For UV-B treatment, the plates were exposed to UV-B irradiation at 1350 mW/m^2^ for 1 h, followed by incubation at 28 °C for 20 h. For wet-heat treatment, the plates were subjected to a 45 °C water-bath treatment for 6 h and subsequently incubated at 28 °C for 20 h. Nuclear morphology was then observed by DAPI staining as described previously [21,22]: samples were fixed with 4% paraformaldehyde for 15 min, washed with PBS, stained with DAPI (1 μg/mL) for 10 min in the dark, and then examined under a fluorescence microscope. The transcript levels of DNA repair genes (*RAD2*, *RAD3*, *RAD4*, *RAD10*, *RAD14*, *RAD16*, *RAD23* and *RAD25*) and heat shock protein genes (*HSP40_1*, *HSP40_2*, *HSP70_1*, *HSP70_2*, *HSP70_3*, and *HSP90*) were determined by RT-qPCR, following the protocol described in Section 2.7.

#### 2.6.3. Detection of ROS Levels and Antioxidant Enzyme Gene Expression

Spore suspensions of the WT, Δ*MaMHR*, and CP strains were aseptically prepared to a final concentration of 1 × 10^7^ spores/mL. Aliquots of 100 µL of each suspension were spread onto 1/4 SDAY plates. Following exposure to UV-B irradiation for 1 h or treatment in a 45 °C water bath for 6 h, the plates were incubated at 28 °C for an additional 20 h before sampling. The spores were then scraped off and incubated with the DCFH-DA probe (final concentration, 10 µmol/L) in the dark at 28 °C for 30 min [23]. Fluorescence microscopy was subsequently used for observation and image acquisition. Additionally, RNA was extracted from another set of identically treated samples, and the transcript levels of antioxidant enzyme genes (*SOD_1*, *SOD_2*, *SOD_4*, *CAT_1*, *CAT_2*, *CAT_3*, *CAT_4*, *POD*, *GPX*, *MnSOD*) were assessed by RT-qPCR as described in Section 2.7.

#### 2.6.4. Determination of Antioxidant Enzyme Activities

The activities of superoxide dismutase (SOD), catalase (CAT), peroxidase (POD), and glutathione peroxidase (GPX) were determined using the corresponding commercial assay kits (Solarbio, Beijing, China): SOD (BC0175), CAT (BC0205), POD (BC0090), and GPX (BC1195), according to the manufacturer’s instructions. Similar kits were used in previous studies [23,24]. Samples collected after the corresponding stress treatment and recovery period were used for enzyme activity assays. Enzyme activities were normalized according to total protein content and expressed as U/mg protein.

#### 2.6.5. Determination of Trehalose Content

Trehalose was extracted and quantified according to the instructions provided by the trehalose content determination kit [25]. Mature spores (0.1 g) from each strain were mixed with 1 mL extraction buffer and disrupted by ultrasonication (200 W, 3 s on/10 s off, 30 cycles). After centrifugation, the supernatant was collected. Trehalose standards were prepared in distilled water at concentrations of 0, 0.003125, 0.00625, 0.0125, 0.025, 0.05, and 0.1 mg/mL. Then, 0.25 mL of each standard or sample was mixed with 1 mL working solution, heated at 95 °C for 10 min, cooled to room temperature, and the absorbance was measured at 620 nm. The standard curve was constructed by plotting ΔA (ΔA = A_standard_ − A_blank_) against the standard concentration, and the trehalose content in the samples was calculated accordingly.

### 2.7. RT-qPCR

Samples collected for gene-expression analyses in Section 2.6.1, Section 2.6.2 and Section 2.6.3 were used for RNA extraction, were ground in liquid nitrogen, and total RNA was extracted using TRIzol reagent. After DNase I digestion to remove genomic DNA, cDNA was synthesized using Oligo(dT) primers. RT-qPCR was performed with SYBR Green premix, using the *M. acridum* GPDH gene as the internal reference. Each 20-µL reaction contained 10 µL of 2 × SYBR Premix, 0.5 µL each of forward and reverse primers (10 µmol/L), 1 µL cDNA, and nuclease-free water to volume. The cycling protocol was: 95 °C for 3 min, followed by 40 cycles of 95 °C for 15 s, 60 °C for 30 s, and 72 °C for 30 s. Relative gene expression was calculated using the 2^−ΔΔCt^ method [23]. All experiments were performed with three biological replicates, each with three technical replicates.

### 2.8. Virulence Assay

#### 2.8.1. Virulence Bioassay

(1) Topical inoculation—Spore suspensions of the WT, Δ*MaMHR*, and CP strains were prepared in liquid paraffin at 1.0 × 10^7^ conidia/mL. A 5-µL droplet of each suspension was applied to the pronotum of each insect; control insects received an equal volume of liquid paraffin.

(2) Intra-hemocoel injection—Spore suspensions (1 × 10^6^ conidia/mL) were prepared in sterile water, and 5 µL of each suspension was injected into the intersegmental membrane between the second and third abdominal segments using a microsyringe. Control insects were injected with an equal volume of sterile water.

Bioassays were performed using fifth-instar nymphs of *Locusta migratoria*. For each strain or control group, 30 nymphs were maintained per cage, and three independent replicates were performed. All treated insects were reared at 28 °C under a 14 h light: 10 h dark photoperiod, with fresh food provided every 12 h. Mortality was recorded every 12 h. Survival curves were generated using the Kaplan–Meier method, and the median lethal time (LT_50_) with 95% confidence intervals was calculated. This method is widely used in entomopathogenic fungal virulence studies [23,26]. The Kaplan–Meier method was chosen for survival analysis because it considers the entire mortality curve across all time points rather than mortality at a specific time point, providing a more comprehensive assessment of fungal virulence. This method is widely used in entomopathogenic fungal virulence studies and allows for comparison of survival distributions using the log-rank test.

#### 2.8.2. Cuticle Penetration Assay

Spore suspensions of the WT, Δ*MaMHR*, and CP strains were prepared at 1 × 10^7^ conidia/mL in 0.05% Tween-80. Sterilised hindwings of L. migratoria were placed onto 1/4 SDAY medium, and 2-µL droplets of each suspension were inoculated onto the wings. After incubation at 28 °C for 2 days, photographs were taken; the wings were then removed, and the plates were further incubated at 28 °C for 4 days before a second set of photographs was recorded. Cuticle penetration assay was performed as described previously [17].

### 2.9. Data Processing

The numbers of biological and technical replicates are indicated in the corresponding experimental procedures and figure legends. Data are presented as mean ± standard deviation (SD). Statistical analyses were performed using GraphPad Prism 9.5.0. One-way ANOVA followed by Tukey’s post-hoc test was used for comparisons among three strains where appropriate. Survival curves from virulence assays were compared using the log-rank (Mantel–Cox) test, and LT_50_ values with 95% confidence intervals were estimated using Kaplan–Meier analysis. IT_50_ values were estimated from the corresponding germination/inactivation curves. Significance levels were defined as * *p* < 0.05, ** *p* < 0.01, *** *p* < 0.001; ns, not significant.

## 3. Results

### 3.1. Phylogenetic and Structural Analysis of MaMHR

A gene encoding a Zn_2_Cys_6_-type transcription factor (MAC_05848) was identified in the *Metarhizium acridum* genome. Domain analysis revealed that the predicted protein contains both a GAL4-like Zn_2_Cys_6_ DNA-binding domain and a fungal transcription factor middle homology region (fungal_TF_MHR) domain; therefore, it was designated *MaMHR* (Figure 1A).

*MaMHR* is 2943 bp in length without introns and encodes a protein of 582 amino acids. The predicted molecular weight and theoretical isoelectric point (pI) are 64.75 kDa and 6.74, respectively.

Phylogenetic analysis and multiple sequence alignment indicated that *MaMHR* is conserved among the examined fungal homologs (Figure 1B). The N-terminal GAL4-like Zn_2_Cys_6_ domain contains conserved cysteine residues and characteristic DNA-binding motifs responsible for Zn^2+^ coordination and DNA binding. The phylogenetic tree showed that *MaMHR* clustered closely with homologs from other Metarhizium species, with high bootstrap support (Figure 1C).

### 3.2. Deletion of MaMHR Delays Conidial Germination and Reduces Conidiation

To investigate the role of *MaMHR* in conidial development in *Metarhizium acridum*, conidial germination and conidial yield were assessed in WT, Δ*MaMHR*, and complemented (CP) strains on 1/4 SDAY medium.

Compared with WT and CP strains, the Δ*MaMHR* mutant exhibited a delayed conidial germination phenotype (Figure 2A), accompanied by a significant increase in germination time (GT_50_) (Figure 2B). In addition, the Δ*MaMHR* mutant showed a significantly reduced conidial yield compared with WT and CP strains (Figure 2C,D).

### 3.3. Deletion of MaMHR Shifts the Conidiation Pattern from Microcycle to Normal Conidiation

To determine whether *MaMHR* regulates conidiation patterns, we examined WT, Δ*MaMHR*, and CP strains on 1/4 SDAY and SYA media.

On SYA medium, WT and CP strains exhibited a typical microcycle conidiation pattern, with conidia appearing at approximately 16 h and rapidly increasing after 24 h. In contrast, the Δ*MaMHR* mutant showed a shift to a normal conidiation pattern, with a delayed onset of conidiation occurring after 24 h (Figure 3A).

On 1/4 SDAY medium, WT and CP strains initiated conidiation at approximately 20 h, with abundant conidia observed at 36 h. The Δ*MaMHR* mutant displayed a delayed initiation of conidiation (after 36 h), while no obvious alteration in conidiation morphology was observed (Figure 3B).

### 3.4. Deletion of MaMHR Reduces Tolerance to UV-B and Heat Shock in Metarhizium acridum

To investigate the role of *MaMHR* in environmental stress responses, the tolerance of WT, Δ*MaMHR*, and complemented (CP) strains to UV-B irradiation and wet heat shock was evaluated based on conidial germination.

Following UV-B exposure, the Δ*MaMHR* mutant generally showed lower germination rates than WT and CP strains, with significant differences observed at several exposure times. In addition, the 50% inhibition time (IT_50_) of the Δ*MaMHR* mutant was significantly shortened (Figure 4A,B).

Similarly, under 45 °C heat shock treatment, the Δ*MaMHR* mutant showed reduced germination, with significant differences observed at several exposure times, and its IT_50_ was significantly lower than those of the WT and CP strains (Figure 4C,D).

### 3.5. Deletion of MaMHR Affects Tolerance to Chemical Stresses in Metarhizium acridum

To evaluate the role of *MaMHR* in chemical stress responses, WT, Δ*MaMHR*, and complemented (CP) strains were cultured on 1/4 SDAY medium supplemented with sorbitol (SOR), NaCl, H_2_O_2_, sodium dodecyl sulfate (SDS), Calcofluor White (CFW), or Congo red (CR), and colony diameter and relative growth rate were measured.

No significant differences in colony growth were observed among WT, Δ*MaMHR*, and CP strains under standard 1/4 SDAY conditions (Figure 5A).

Under SOR, CFW, and CR treatments, the Δ*MaMHR* mutant exhibited significantly reduced colony diameter and relative growth rate compared with WT and CP strains. Under SDS treatment, the Δ*MaMHR* mutant showed a slight reduction in colony growth, which was not statistically significant (Figure 5B,C). These results indicate that *MaMHR* deletion primarily impairs tolerance to cell wall integrity (CFW, CR) and osmotic (SOR) stresses, rather than oxidative (H_2_O_2_) or ionic (NaCl) stresses.

### 3.6. Deletion of MaMHR Reduces Melanin Production in Metarhizium acridum

To investigate the role of *MaMHR* in the regulation of protective metabolite accumulation, we first examined melanin production in WT, Δ*MaMHR*, and complemented (CP) strains. The conidial suspension of the Δ*MaMHR* mutant appeared visibly lighter in pigmentation compared with those of WT and CP strains (Figure 6A). Quantitative spectrophotometric analysis further confirmed that melanin content was significantly reduced in the Δ*MaMHR* mutant, whereas complementation restored melanin levels to those of the WT strain (Figure 6B).

To explore the underlying molecular mechanism, we examined the transcriptional levels of multiple melanin biosynthesis-related genes by RT-qPCR. The expression of PksP, SCD, THR, Abr1, Tyr, HppD, and LAC was significantly downregulated in the Δ*MaMHR* mutant compared with WT, while complementation restored their expression to wild-type levels (Figure 6C). These results suggest that *MaMHR* contributes to melanin biosynthesis and is associated with the transcriptional expression of melanin biosynthesis-related genes.

In contrast to the pronounced effects on melanin, we measured trehalose content in mature conidia of the three strains to determine whether *MaMHR* also participates in trehalose-mediated stress defense. As shown in Figure 6D, no significant differences in trehalose content were observed among WT, Δ*MaMHR*, and CP strains, suggesting that *MaMHR* does not contribute to trehalose accumulation under the conditions tested. Collectively, these findings suggest that *MaMHR* contributes to melanin accumulation, whereas trehalose content was not significantly altered under the conditions tested.

### 3.7. Deletion of MaMHR Affects ROS Accumulation and Antioxidant Responses Under UV-B and Heat Shock

To investigate the role of *MaMHR* in oxidative stress responses, intracellular ROS accumulation and antioxidant system activities were analyzed in WT, Δ*MaMHR*, and complemented (CP) strains following UV-B or heat shock.

DCFH-DA staining revealed detectable ROS-associated fluorescence in all strains after 20 h post-treatment incubation following UV-B or heat shock. Qualitative fluorescence microscopy revealed that the Δ*MaMHR* mutant exhibited stronger ROS-associated fluorescence signals than WT and CP (Figure 7A,D).

Given the disruption of internal ROS homeostasis and altered tolerance to environmental stresses, we further evaluated the transcriptional responses of antioxidant-related genes in the mutant. RT-qPCR analysis revealed distinct expression patterns among the antioxidant genes examined. Under UV-B stress, *SOD_1*, *CAT_1*, *CAT_4*, *POD*, and *MnSOD* were significantly upregulated in the Δ*MaMHR* mutant, whereas *GPX*, *SOD_2*, *SOD_4*, *CAT_2*, and *CAT_3* showed no significant differences (Figure 7B). Under heat shock, GPX, *SOD_1*, *SOD_4*, *CAT_2*, *CAT_3*, *POD*, and *MnSOD* were significantly upregulated in the Δ*MaMHR* mutant, whereas CAT1 and CAT4 showed no significant differences (Figure 7E). These results suggest that deletion of *MaMHR* alters the transcriptional responses of multiple antioxidant-related genes under environmental stress.

Furthermore, we measured the activities of antioxidant enzymes under stress conditions. Following UV-B treatment, GPX and CAT activities were significantly elevated in the Δ*MaMHR* mutant, whereas the activities of SOD and POD showed no significant differences (Figure 7C). Following heat treatment, GPX, CAT, and POD activities were significantly increased in the Δ*MaMHR* mutant, whereas SOD activity showed no significant difference (Figure 7F). The discrepancy between transcript levels and enzymatic activities suggests that transcriptional changes do not fully account for the observed antioxidant responses. Together with the stronger ROS-associated fluorescence observed in the mutant, these results indicate that *MaMHR* deletion is associated with altered antioxidant responses under both UV-B and heat shock.

### 3.8. Deletion of MaMHR Affects Nuclear Morphology and DNA Repair- and Heat Shock-Related Gene Expression Under UV-B and Heat Shock

DAPI staining revealed that, after UV-B or heat shock, nuclei in the Δ*MaMHR* mutant exhibited dispersed and irregular morphology compared with the intact, round nuclei observed in WT and CP strains (Figure 8A,B).

Following UV-B treatment, RT-qPCR analysis further showed that *RAD2*, *RAD3*, *RAD4*, *RAD10*, *RAD14*, and *RAD23* were significantly downregulated in the Δ*MaMHR* mutant, whereas RAD16 showed no significant difference. *RAD25* transcript levels were significantly upregulated in the mutant, suggesting a potentially compensatory or uncoupled transcriptional response within the repair machinery (Figure 8C). Under wet heat shock, the expression of *HSP40_1* and *HSP70_3* was significantly increased in the Δ*MaMHR* mutant, whereas *HSP40_2* was significantly decreased; *HSP70_1*, *HSP70_2*, and *HSP90* showed no significant differences among the three strains (Figure 8D).

### 3.9. Deletion of MaMHR Reduces Topical Virulence but Not Virulence Following Intra-Hemocoel Injection

To evaluate the role of *MaMHR* in pathogenicity, virulence bioassays were performed against fifth-instar nymphs of Locusta migratoria via topical inoculation, intra-hemocoel injection, and cuticle penetration assays.

Following topical inoculation, the Δ*MaMHR* mutant exhibited significantly reduced virulence compared with the WT and CP strains, as reflected by a prolonged median lethal time (LT_50_) (*p* < 0.01; Figure 9A,C). Consistently, the cuticle penetration assay showed that the Δ*MaMHR* mutant displayed markedly reduced fungal outgrowth after hindwing removal, suggesting a defect in cuticle penetration-associated fungal development (Figure 9E).

In contrast, when the fungus was directly introduced into the hemocoel via intra-hemocoel injection, no significant difference in mortality was observed among the WT, Δ*MaMHR*, and CP strains (Figure 9B,D), indicating that the virulence defect observed after topical inoculation is largely bypassed following direct entry into the host hemocoel. These results suggest that the major defect occurs during early infection, particularly in processes associated with host cuticle penetration.

## 4. Discussion

Environmental adaptation is essential for the survival and biocontrol efficiency of entomopathogenic fungi, particularly under field conditions where conidia are frequently exposed to UV radiation, temperature fluctuations, and other environmental stresses [20,21]. In this study, we identified *MaMHR* as an MHR-containing Zn_2_Cys_6_ transcription factor that contributes to developmental regulation, stress adaptation, and pathogenicity in *Metarhizium acridum*. Unlike many previously characterized Zn_2_Cys_6_ transcription factors involved in individual metabolic or developmental processes, *MaMHR* is involved in multiple physiological processes, including conidiation [14,21], melanin production [22], oxidative homeostasis, DNA damage responses, and host infection. The fungal-specific middle homology region (MHR) has been proposed to contribute to transcriptional regulation and protein interactions, yet its biological significance in entomopathogenic fungi remains poorly understood [16].

Deletion of *MaMHR* caused delayed conidial germination, reduced conidial production, and a transition from microcycle conidiation to normal conidiation, indicating that *MaMHR* participates in regulating fungal developmental programs. Similar developmental regulation has been reported for other transcription factors in *M. acridum*, including MaNCP1 and MaMsn2, which influence conidiation pattern switching through distinct regulatory pathways. However, the involvement of an MHR-containing Zn_2_Cys_6_ transcription factor in conidiation regulation has not been previously described in entomopathogenic fungi.

Oxidative stress is one of the major acute host and environmental challenges for entomopathogenic fungi, where excessive accumulation of reactive oxygen species (ROS) causes irreversible cellular damage. In the present study, the Δ*MaMHR* mutant exhibited elevated endogenous ROS accumulation following exposure to UV-B radiation and heat shock [24]. Intriguingly, the mutant displayed unaltered sensitivity to exogenous H_2_O_2_. This phenomenon suggests a distinct, stimulus-specific regulatory role for *MaMHR* in fungal redox homeostasis. Exogenous H_2_O_2_ stress represents an acute chemical shock primarily counteracted by cell wall/membrane-associated detoxification barriers and basal antioxidant enzymes. In contrast, physical environmental stressors such as UV-B and wet heat trigger intrinsic organelle dysfunction (e.g., in mitochondria) and sustained endogenous ROS generation. Thus, *MaMHR* may contribute to the regulation of intracellular ROS homeostasis during physical stress adaptation rather than serving as a general responder to external oxidative agents. However, whether *MaMHR* itself is transcriptionally induced by environmental stress remains to be determined. Furthermore, although antioxidant enzymes (such as *CAT* and *GPX*) were transcriptionally up-regulated or showed increased enzymatic activities in the Δ*MaMHR* strain under UV-B and heat treatments, this compensatory response was insufficient to restore internal redox balance. This imbalance, combined with reduced protective melanin accumulation and impaired DNA repair gene expression, collectively exacerbates the sensitivity of the Δ*MaMHR* mutant to physical environmental stresses [23].

The concurrent increase in ROS-associated fluorescence and antioxidant enzyme activities in the *MaMHR* mutant suggests the activation of a cellular compensatory response to oxidative stress [25,26,27]. Although the mutant showed increased transcription of several antioxidant genes, this response was insufficient to restore redox homeostasis. Additionally, its unaltered sensitivity to exogenous H_2_O_2_ reveals that *MaMHR* is more closely associated with intracellular ROS homeostasis under developmental or environmental stress conditions than with responses to exogenous H_2_O_2_.

Melanin represents an important protective barrier against environmental stresses in many filamentous fungi by absorbing ultraviolet radiation and reducing oxidative damage. In the present study, deletion of *MaMHR* significantly decreased melanin accumulation and downregulated the expression of multiple melanin biosynthesis genes, suggesting that *MaMHR* positively contributes to melanin production. Reduced melanin accumulation is likely to weaken the physical protection of conidia against UV irradiation, thereby increasing susceptibility to environmental stress [27]. Consistent with this hypothesis, the Δ*MaMHR* mutant also exhibited abnormal nuclear morphology after UV-B and heat treatment, accompanied by reduced expression of several genes involved in the nucleotide excision repair (NER) pathway. As NER is the major pathway responsible for repairing UV-induced DNA lesions, decreased expression of these genes may be associated with altered DNA damage responses following UV-B stress. Therefore, reduced melanin accumulation together with altered DNA damage-associated responses may contribute to the increased stress sensitivity observed in the mutant [28,29].

Developmental processes in entomopathogenic fungi are closely associated with intracellular redox homeostasis. In this study, deletion of *MaMHR* resulted in delayed conidial germination, reduced conidial production, and a transition from microcycle conidiation to normal conidiation. These developmental defects were accompanied by disturbances in ROS homeostasis and DNA damage responses, suggesting that maintenance of intracellular redox balance and genome stability is required for normal conidial development. Interestingly, trehalose content was not significantly altered in the Δ*MaMHR* mutant, indicating that the reduced stress tolerance caused by *MaMHR* deletion is unlikely to be mediated through trehalose metabolism. Instead, alterations in oxidative stress regulation, melanin biosynthesis, and DNA damage response-related gene expression may contribute to the *MaMHR*-dependent stress phenotype.

The virulence assays further revealed that *MaMHR* is specifically associated with fungal infection via the topical/cuticular route, whereas direct injection into the hemocoel bypassed the observed defect [29,30,31]. These results suggest that *MaMHR* contributes to the early infection process, particularly cuticle penetration-associated growth. The reduced cuticle penetration-associated fungal growth is consistent with the reduced stress tolerance and pigmentation defects observed in the Δ*MaMHR* mutant, as successful infection initiation requires fungal adaptation to external stresses encountered during host surface colonization. Given that *MaMHR* affects melanin biosynthesis and oxidative stress responses, these defects may contribute to impaired fungal development during the early stages of host infection. Notably, the unchanged virulence upon hemocoel injection indicates that the *MaMHR*-dependent defect observed during topical infection is largely bypassed after direct entry into the host hemocoel [31,32,33].

No additional *M. acridum* protein containing both a Zn_2_Cys_6_ DNA-binding domain and an MHR domain was identified in our sequence analysis, suggesting that *MaMHR* is the only recognizable MHR-containing Zn_2_Cys_6_ transcription factor in the current genome assembly. However, the possibility of functional compensation by other Zn_2_Cys_6_ transcription factors cannot be excluded. Such functional redundancy could contribute to the relatively subtle phenotypic differences observed between the WT and Δ*MaMHR* mutant in certain assays. Further systematic characterization of the Zn_2_Cys_6_ transcription factor family in *M. acridum* will be required to determine whether other family members contribute to the phenotypes observed in the Δ*MaMHR* mutant.

Based on these findings, we propose that *MaMHR* is associated with oxidative stress responses, melanin biosynthesis, and DNA repair-associated processes in *M. acridum*. Loss of *MaMHR* disrupts ROS homeostasis, reduces melanin accumulation, and alters the expression of DNA repair-related genes, which may contribute to impaired conidial development and reduced tolerance to UV-B and wet heat shock. Future studies should focus on identifying the direct downstream targets of *MaMHR* and elucidating the transcriptional regulatory network through which this Zn_2_Cys_6_ transcription factor controls fungal development and environmental adaptation.

Several limitations of this study should be acknowledged. First, although our phenotypic analyses clearly demonstrate that *MaMHR* deletion affects conidiation, stress tolerance, and virulence, we have not yet established the temporal and spatial expression profile of *MaMHR* across different developmental stages (e.g., vegetative mycelium, conidiation, conidial germination, and early host infection) in the wild-type strain. Such data would further clarify whether *MaMHR* functions as a constitutive regulator or is developmentally stage-specific. Second, whether *MaMHR* transcript levels are directly induced by environmental stressors (UV-B, heat shock, sorbitol, or CFW) remains unknown. Without this information, we cannot fully exclude the possibility that some of the stress-sensitive phenotypes observed in the Δ*MaMHR* mutant are partially secondary consequences of overall reduced cellular fitness, although the specific alterations in melanin and ROS pathways argue for a more direct role. Third, while our genome-wide BLASTP search using both the Zn_2_Cys_6_ and MHR domains did not identify additional paralogs, no additional *M. acridum* protein containing both a Zn_2_Cys_6_ DNA-binding domain and an MHR domain was identified in our sequence analysis, suggesting that *MaMHR* is the only recognizable MHR-containing Zn_2_Cys_6_ transcription factor in the current genome assembly. Finally, the present study primarily relied on in vitro and ex vivo assays; further in vivo transcriptional analysis during the natural infection process on host cuticle would provide deeper insights into the precise regulatory role of *MaMHR* in pathogenesis.

## 5. Conclusions

In conclusion, this study identified *MaMHR* as a previously uncharacterized Zn_2_Cys_6_ transcription factor containing a fungal transcription factor middle homology region (MHR) in *Metarhizium acridum*. Functional analyses demonstrated that deletion of *MaMHR* delayed conidial germination, reduced conidial production by approximately 17.6% and 28.3% on 1/4 SDAY and SYA media, respectively, and altered the conidiation pattern from microcycle conidiation to normal conidiation. Furthermore, topical virulence was reduced, as reflected by an approximately 14.6% increase in LT_50_, whereas virulence following intra-hemocoel injection was not significantly affected.

At the molecular level, loss of *MaMHR* reduced melanin content by approximately 47.0% (Figure 6B), significantly downregulated the expression of seven melanin biosynthesis-related genes (Figure 6C), and decreased UV-B and heat shock IT_50_ by 26.5% and 22.6%, respectively (Figure 4B,D), whereas trehalose content remained unchanged (Figure 6D). These findings suggest that *MaMHR* contributes to stress tolerance through effects on oxidative stress regulation, melanin biosynthesis, and DNA repair-associated responses, whereas trehalose accumulation is unlikely to be a major contributor under the conditions tested.

Overall, this study identifies a previously unrecognized role of the MHR-containing Zn_2_Cys_6_ transcription factor *MaMHR* in coordinating fungal development and environmental adaptation in *M. acridum*. Given that cuticle penetration and solar UV-B tolerance are important determinants of mycoinsecticide performance in the field, our findings suggest that *MaMHR* may warrant further investigation as a candidate for genetic engineering aimed at improving stress tolerance and biocontrol performance.

## Figures and Tables

**Figure 1:**
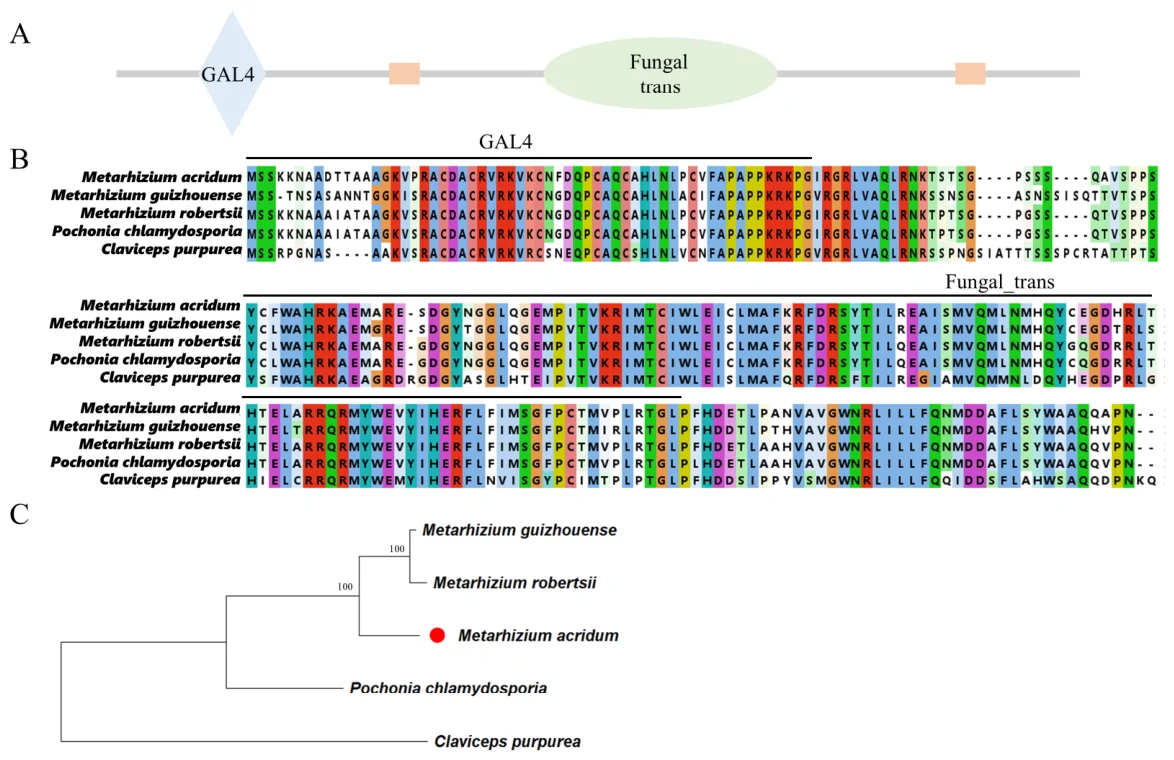
Sequence Analysis and Phylogenetic Tree Construction of *MaMHR*. (**A**) Domain architecture of *MaMHR*. (**B**) Multiple sequence alignment of the GAL4-like Zn_2_Cys_6_ DNA-binding and fungal_TF/MHR regions among selected fungal homologs. The MHR homologous proteins are from *Metarhizium acridum* (XP_007812188.1), *Metarhizium guizhouense* (KID86764.1), *Metarhizium robertsii* (XP_007824950.1), *Pochonia chlamydosporia* (XP_018138382.1), *Claviceps purpurea* (KAG6150444.1). (**C**) Phylogenetic analysis of *MaMHR* proteins in fungi. Bootstrap values (≥50%) are shown at nodes.

**Figure 2:**
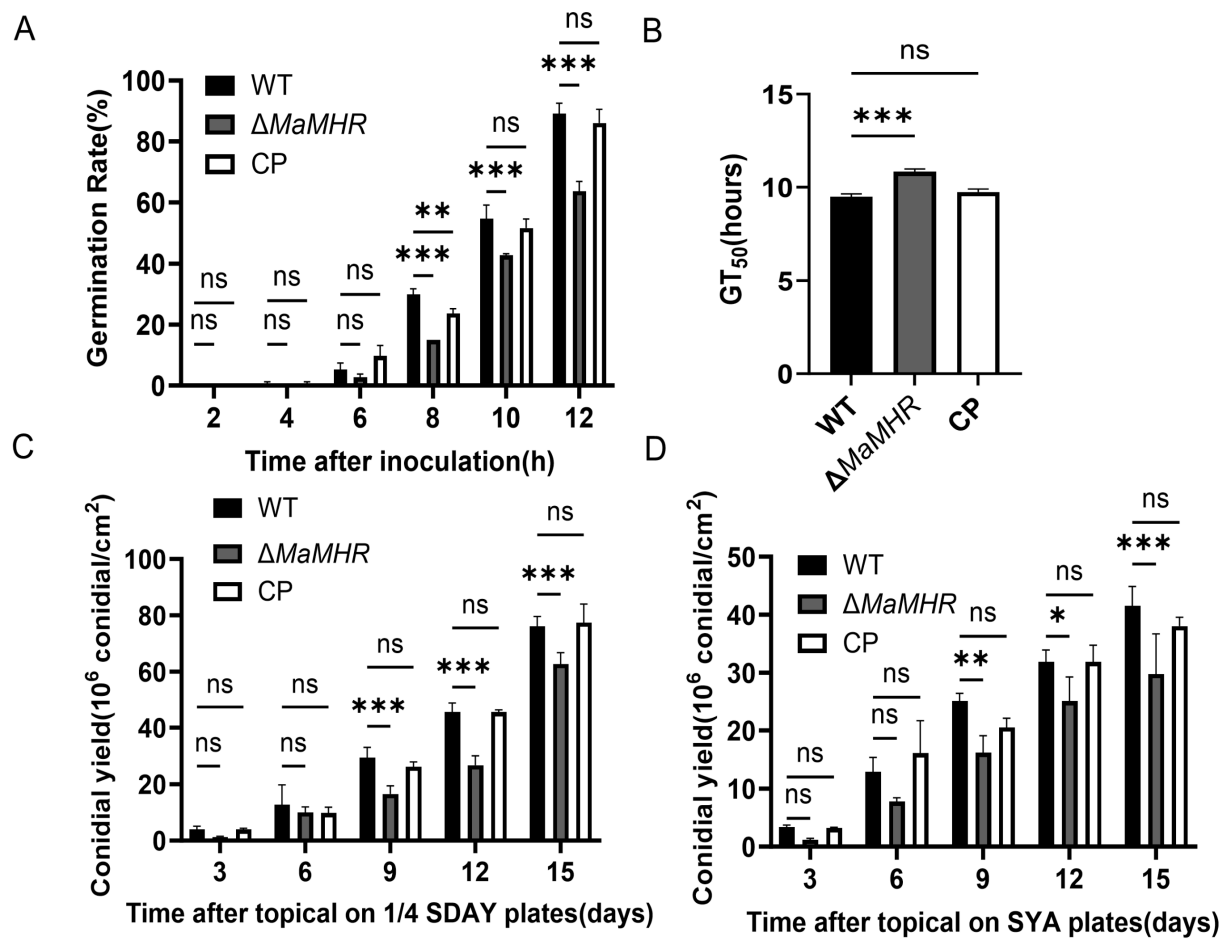
Effects of *MaMHR* deletion on conidial germination and conidial production. (**A**) Conidial germination rates of wild-type (WT), Δ*MaMHR* knockout mutant, and complemented (CP) strains on 1/4 SDAY medium. (**B**) Germination time (GT_50_) of WT, Δ*MaMHR*, and CP strains. (**C**) Conidial production of WT, Δ*MaMHR*, and CP strains on 1/4 SDAY medium. (**D**) Conidial production of WT, Δ*MaMHR*, and CP strains on SYA medium. Data are mean ± SD (n = 3). Significant differences were determined by one-way ANOVA with Tukey’s test (* *p* < 0.05, ** *p* < 0.01, *** *p* < 0.001; ns, not significant).

**Figure 3:**
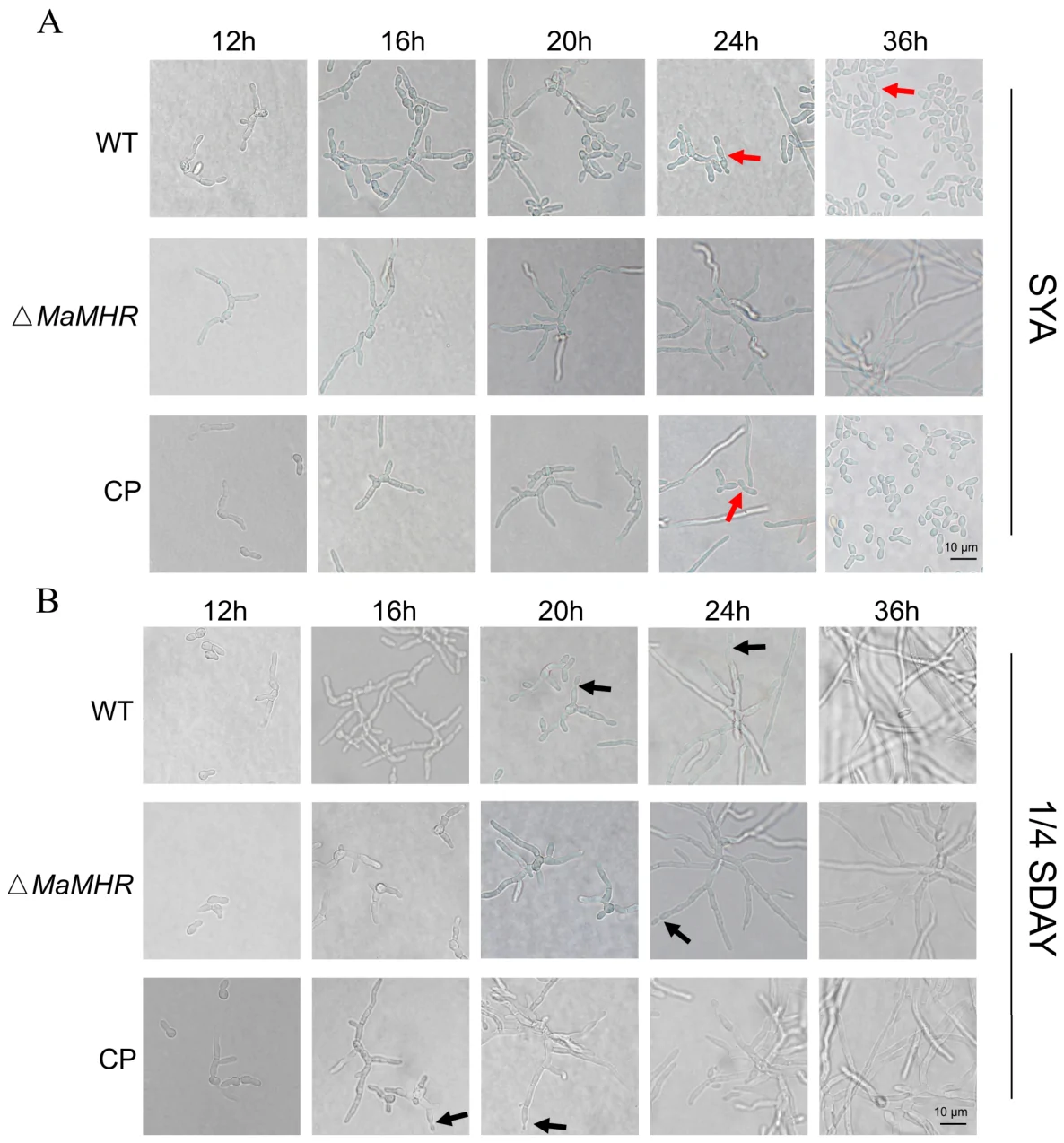
Effects of *MaMHR* on conidiation pattern in *Metarhizium acridum*. (**A**) Conidiation patterns of WT, Δ*MaMHR*, and complemented (CP) strains on SYA medium (microcycle conidiation condition). (**B**) Conidiation patterns of WT, Δ*MaMHR*, and CP strains on 1/4 SDAY medium (normal conidiation condition). Red arrows indicate microcycle conidiation; black arrows indicate normal conidiation. Scale bars = 10 μm.

**Figure 4:**
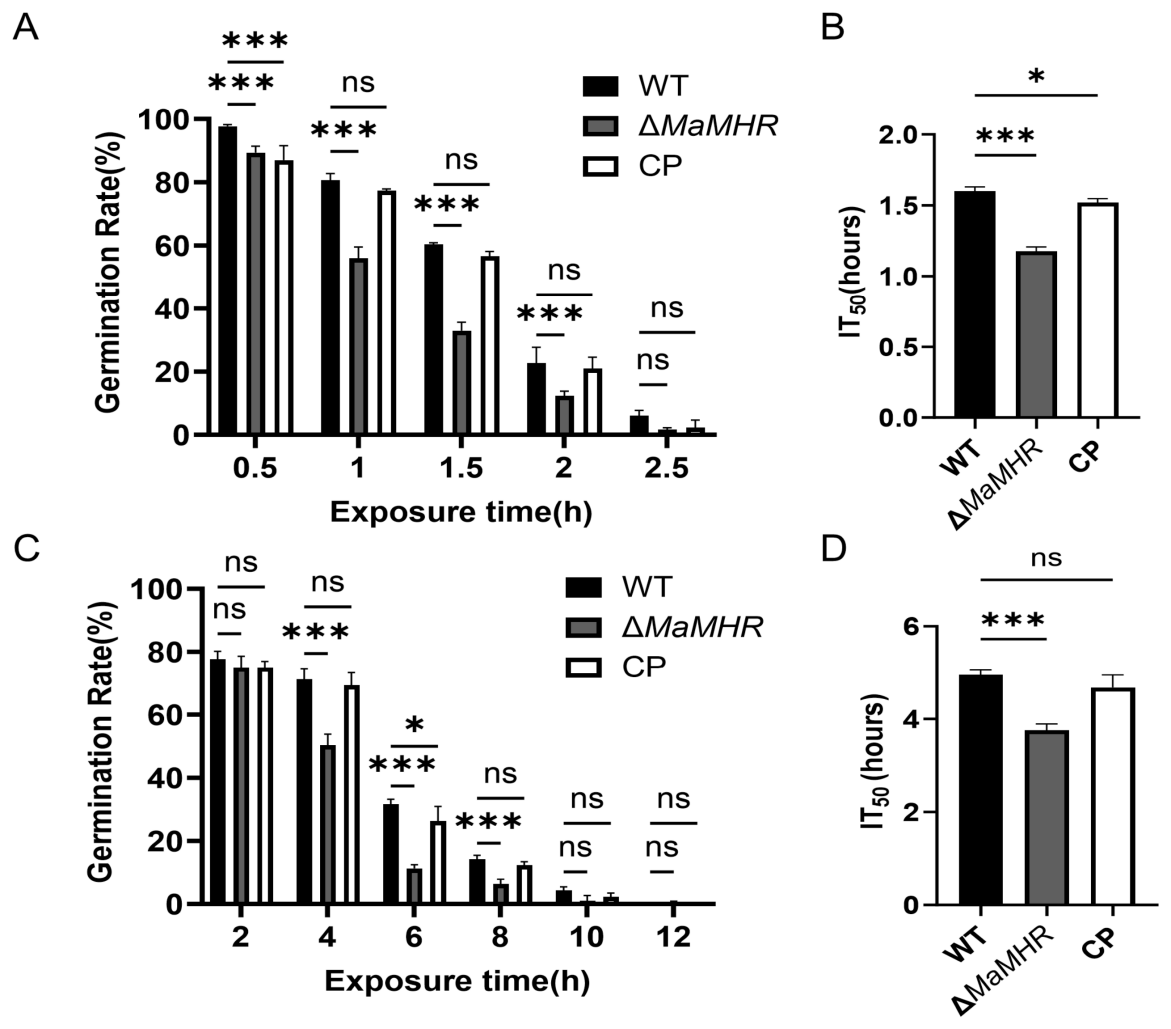
UV-B and heat shock tolerance of wild-type (WT), Δ*MaMHR*, and complemented (CP) strains. (**A**) Conidial germination rate of WT, Δ*MaMHR*, and CP strains after ultraviolet radiation treatment for 0.5, 1, 1.5, 2 and 2.5 h. (**B**) The 50% inhibition time (IT_50_) following UV-B treatment. (**C**) Conidial germination rate of WT, Δ*MaMHR*, and CP strains after heat treatment at 45 °C for 2, 4, 6, 8, 10 and 12 h. (**D**) A 50% inhibition time (IT_50_) following heat shock treatment. Value bars labeled with “ns” represent no significant differences, while asterisks indicate significant differences at *** *p* < 0.001 and * *p* < 0.05.

**Figure 5:**
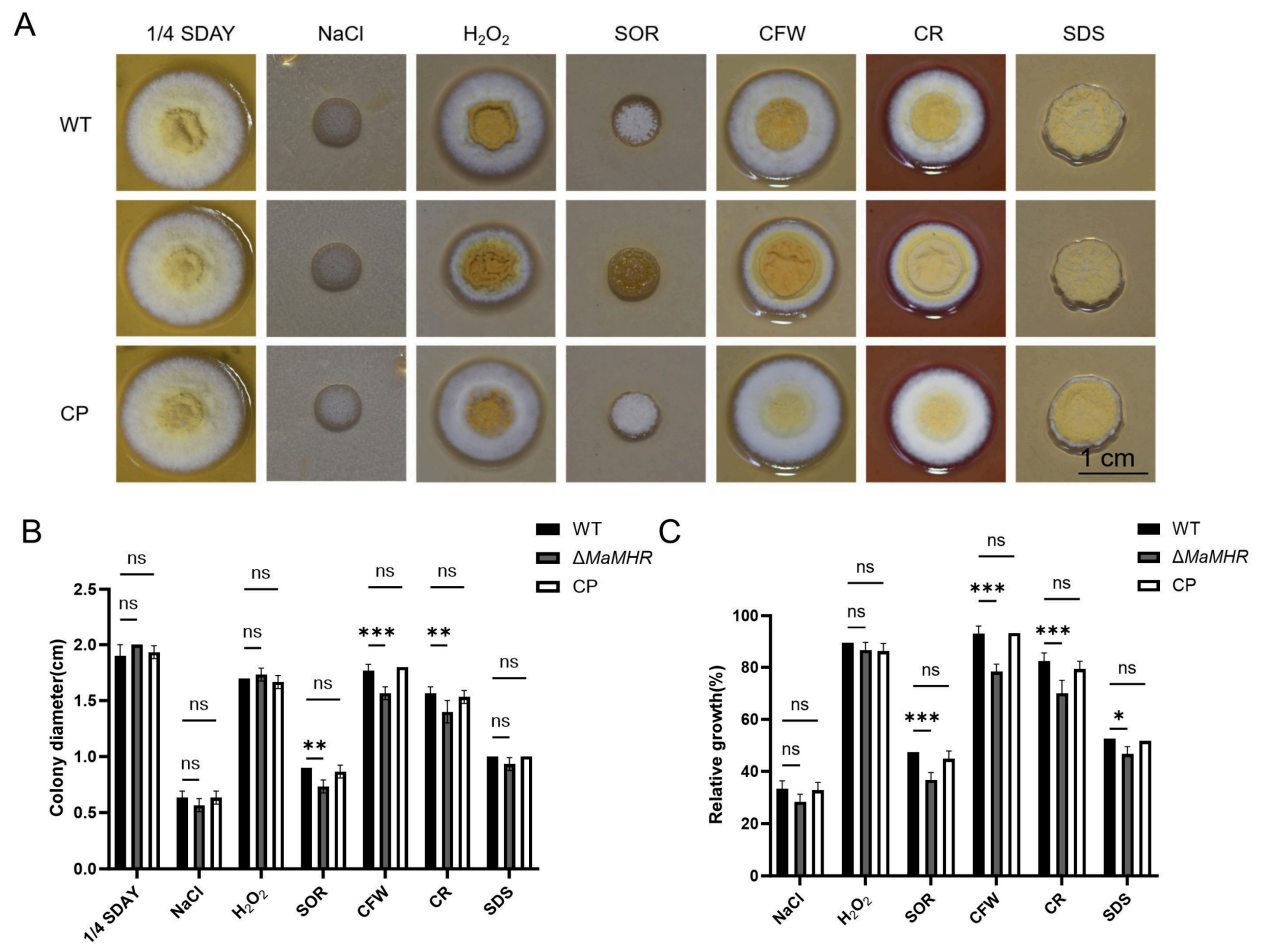
Effects of *MaMHR* deletion on tolerance to chemical stresses. (**A**) Colony morphology of wild-type (WT), Δ*MaMHR*, and complemented (CP) strains on 1/4 SDAY medium supplemented with sorbitol (SOR), sodium chloride (NaCl), hydrogen peroxide (H_2_O_2_), sodium dodecyl sulfate (SDS), Calcofluor White (CFW), or Congo red (CR) for 6 days. (**B**) Colony diameter of fungal strains under different chemical stress conditions. (**C**) Relative growth rate of fungal strains under different chemical stress conditions. Value bars labeled with “ns” represent no significant differences, while asterisks indicate significant differences at *** *p* < 0.001, ** *p* < 0.01, and * *p* < 0.05. Scale bar = 1 cm.

**Figure 6:**
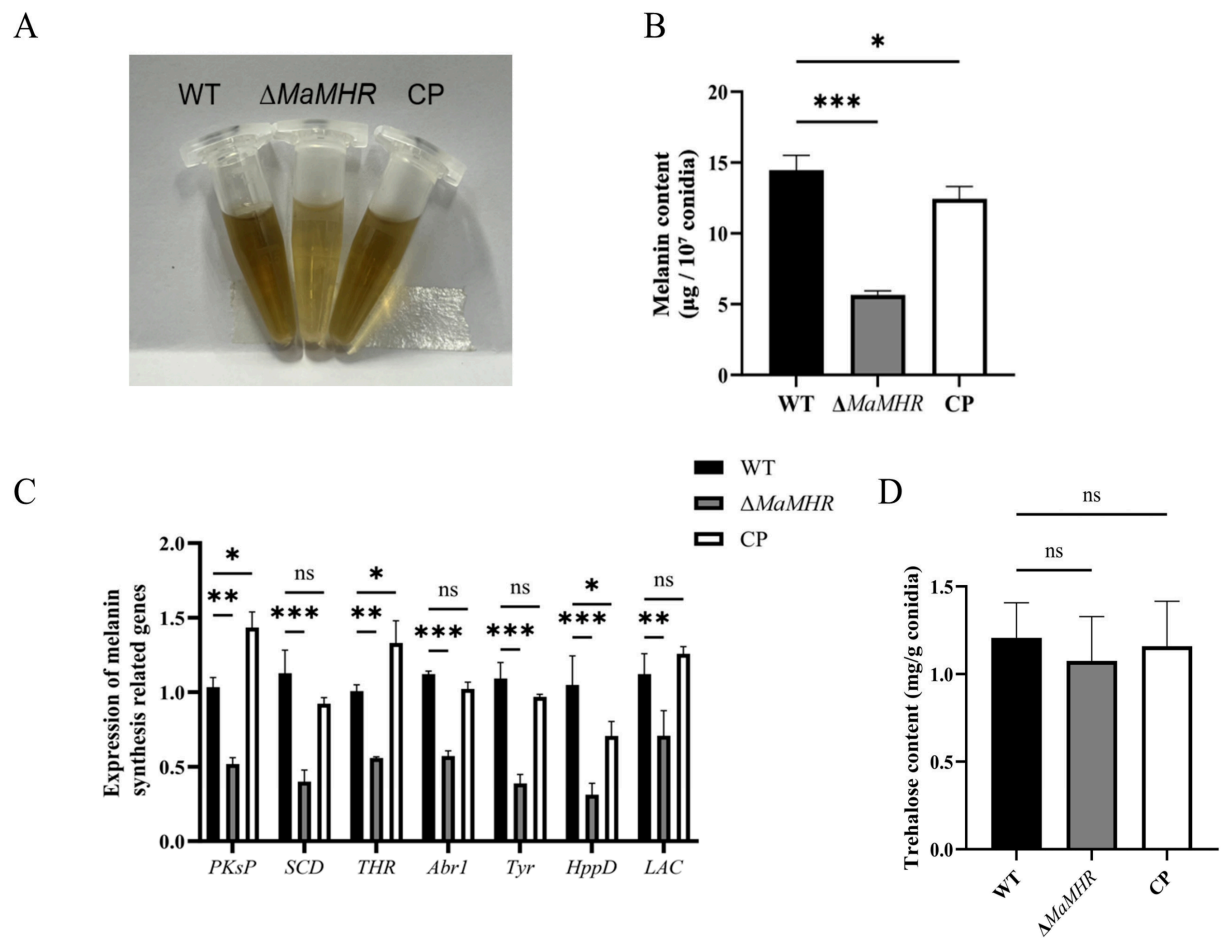
Deletion of *MaMHR* impairs melanin biosynthesis. (**A**) Visual comparison of melanin pigmentation in conidia from 15-day-old cultures of wild-type (WT), Δ*MaMHR*, and complemented (CP) strains. (**B**) Quantitative spectrophotometric analysis of melanin content extracted from conidia. (**C**) Relative transcriptional levels of key genes involved in the DHN-melanin and related pigmentation pathways, including polyketide synthase (PksP), tetrahydroxynaphthalene reductase (THR), scytalone dehydratase (SCD), Abr1, tyrosinase (Tyr), 4-hydroxyphenylpyruvate dioxygenase (HppD), and laccase (LAC), as determined by RT-qPCR. (**D**) Trehalose content in mature conidia of the three strains. Data are presented as mean ± SD (n = 3). Significant differences are indicated by asterisks: *** *p* < 0.001, ** *p* < 0.01, and * *p* < 0.05; ns, not significant (one-way ANOVA with Tukey’s test).

**Figure 7:**
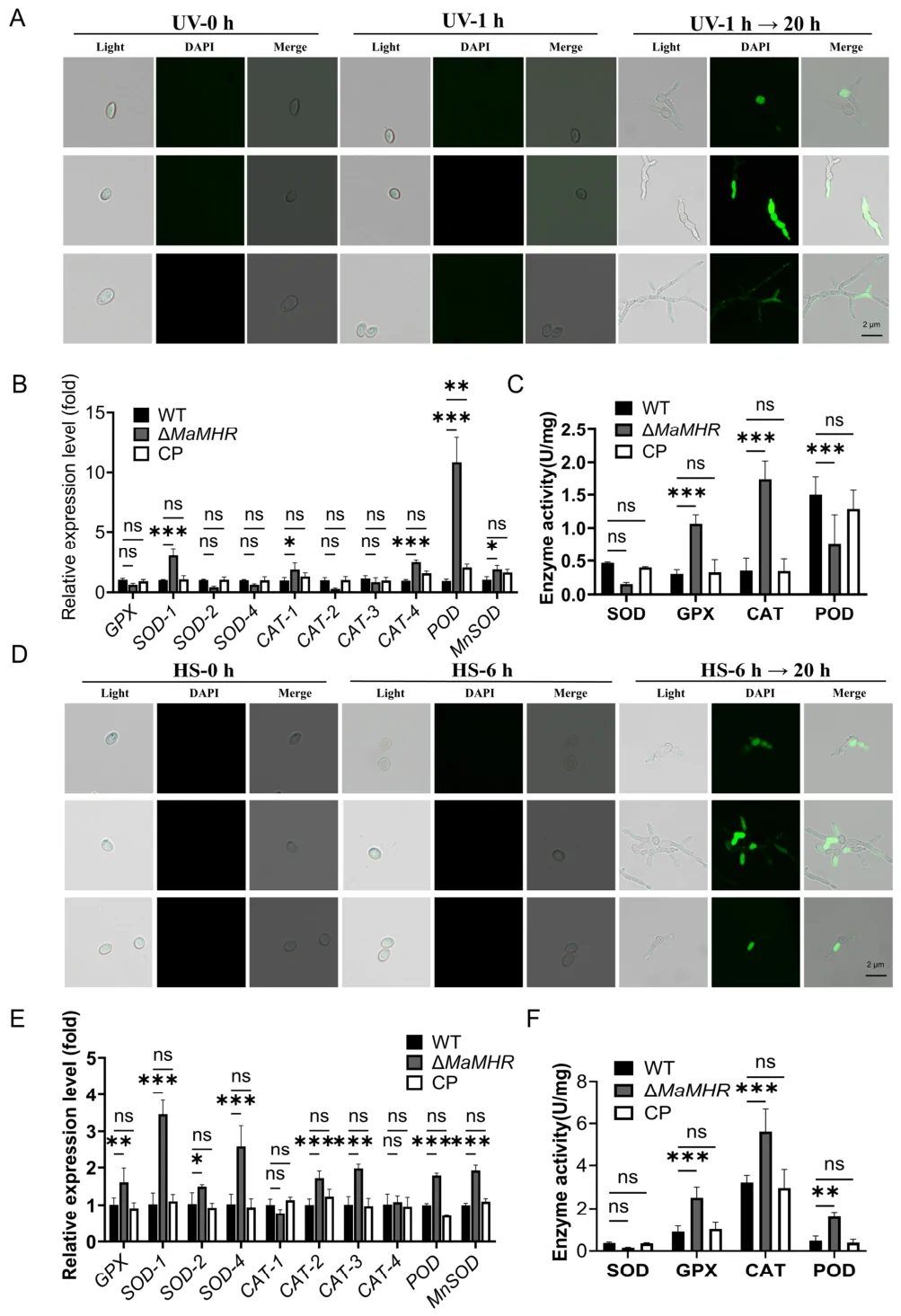
Deletion of *MaMHR* disrupts ROS homeostasis and alters antioxidant responses under UV-B and heat shock. (**A**,**D**) Dichlorodihydrofluorescein diacetate (DCFH-DA) staining showing ROS-associated fluorescence in WT, Δ*MaMHR*, and CP strains after UV-B irradiation for 1 h or heat treatment at 45 °C for 6 h, followed by an additional 20 h incubation at 28 °C. (**B**,**E**) Relative expression of antioxidant-related genes under UV-B or heat shock determined by RT-qPCR. (**C**,**F**) Activities of superoxide dismutase (SOD), catalase (CAT), peroxidase (POD), and glutathione peroxidase (GPX) in wild-type (WT), Δ*MaMHR*, and complemented (CP) strains following UV-B (**C**) or heat (**F**) stress. Data are presented as mean ± SD (n = 3). Significant differences are indicated by asterisks: * *p* < 0.05, ** *p* < 0.01, *** *p* < 0.001; ns, not significant.

**Figure 8:**
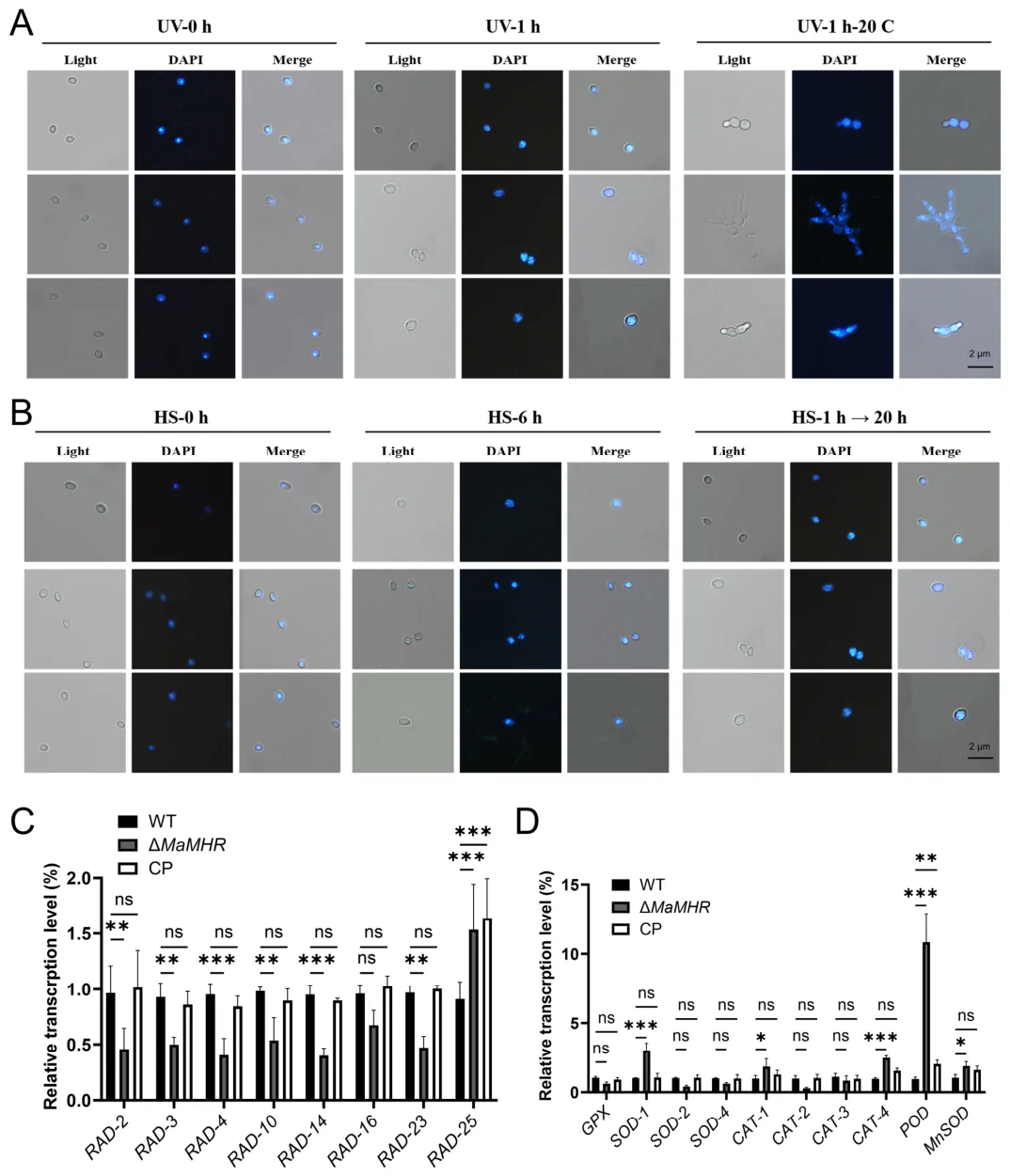
Deletion of *MaMHR* affects nuclear morphology and the expression of DNA repair- and heat shock-related genes in *Metarhizium acridum* following UV-B irradiation and wet heat treatment. (**A**) Nuclear morphology of WT, Δ*MaMHR*, and complemented (CP) after 1 h of UV-B irradiation followed by an additional 20 h incubation at 28 °C. (**B**) Nuclear morphology of wild-type (WT), Δ*MaMHR*, and complemented (CP) strains after heat shock treatment for 6 h followed by an additional 20 h incubation at 28 °C. (**C**) Relative transcript levels of nucleotide excision repair (NER)-related genes (*RAD2*, *RAD3*, *RAD4*, *RAD10*, *RAD14*, *RAD16*, *RAD23* and *RAD25*) following UV-B treatment. (**D**) Relative transcript levels of heat shock protein genes (*HSP40_1*, *HSP40_2*, *HSP70_1*, *HSP70_2*, *HSP70_3*, and *HSP90*) following wet heat treatment. Data are presented as mean ± SD (n = 3). Significant differences are indicated by asterisks: * *p* < 0.05, ** *p* < 0.01, *** *p* < 0.001; ns, not significant.

**Figure 9:**
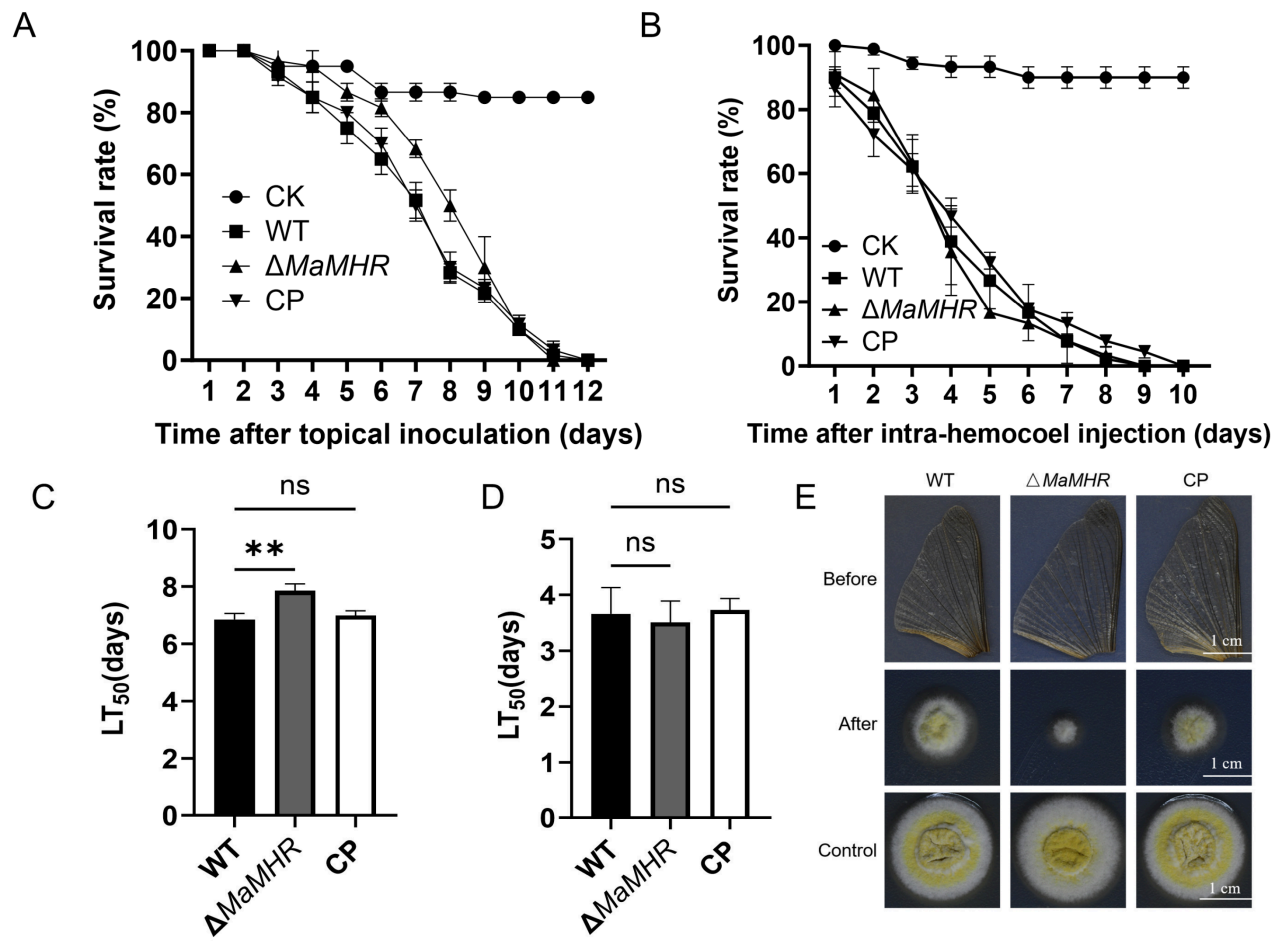
Deletion of *MaMHR* reduces topical virulence and cuticle penetration-associated fungal growth but not virulence following intra-hemocoel injection. (**A**) Survival curves of fifth-instar L. migratoria nymphs after topical inoculation with 5 µL of conidial suspension (1 × 10^7^ conidia/mL) of wild-type (WT), Δ*MaMHR*, and complemented (CP) strains. (**B**) Survival curves after intra-hemocoel injection with 5 µL of conidial suspension (1 × 10^6^ conidia/mL). Liquid paraffin and sterile water were used as blank controls, respectively. (**C**) Median lethal time (LT_50_) for each strain under topical inoculation. (**D**) Median lethal time (LT_50_) for each strain under intra-hemocoel injection. (**E**) Cuticle penetration assay showing hyphal outgrowth from sterilized hindwings inoculated with the three strains. Photographs were taken at 2 days post-inoculation (upper panel) and after wing removal followed by 4 days of further incubation (lower panel). Scale bars = 10 mm. Significant differences are indicated by asterisks: ** *p* < 0.01 and ns, not significant.

## Data Availability

The original contributions presented in this study are included in the article/Supplementary Materials.

## Supplementary Materials

The following supporting information can be downloaded at: https://www.mdpi.com/article/10.3390/jof12100718/s1: Figure S1: The disruption and complementation of *MaMHR*. (A) The knockout and complement schematic diagram of *MaMHR*. (B) PCR was used to verify the complementary strains of *MaMHR*. (C) Transcription-level analysis of *MaMHR* in WT, Δ*MaMHR*, and CP strains. Asterisks indicate significant differences (*** *p* < 0.001; ns, not significant) as determined by one-way ANOVA with Tukey’s test. Table S1. Primers used in this work.
