# Supplementary material for: MaMHR Contributes to Conidiation, Stress Adaptation, and Virulence in Metarhizium acridum
Source: J Fungi (Basel). 2026 Sep 24;12(10):718. doi: 10.3390/jof12100718 (PMC13706419; doi:10.3390/jof12100718)
Supplement: Supplementary file 1 [file jof-12-00718-s001.zip › Figure S1.pdf]

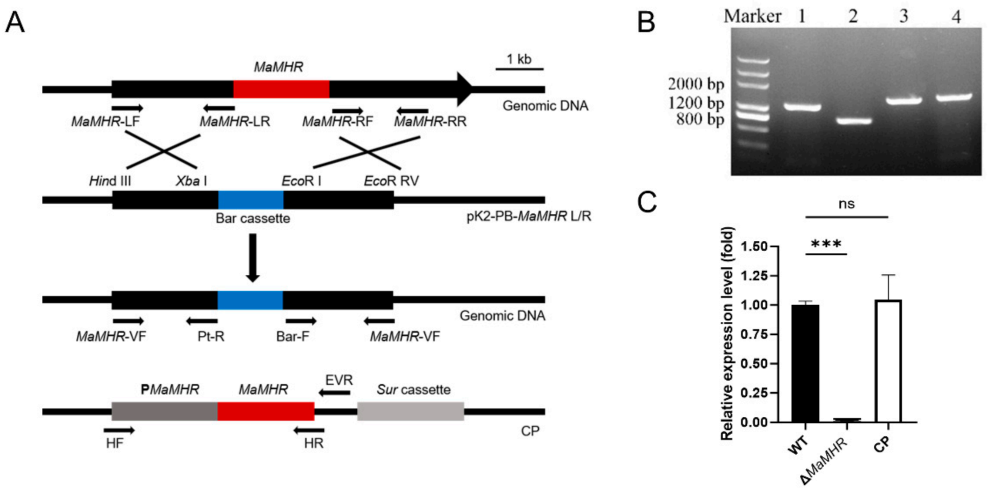

Figure S1: The disruption and complementation of MaMHR. (A) The knockout and complement schematic diagram of MaMHR. (B) PCR was used to verify the complementary strains of MaMHR. (C) Transcription-level analysis of MaMHR in WT, Δ*MaMHR*, and CP strains. Asterisks indicate significant differences (*** $p < 0.001$; ns, not significant) as determined by one-way ANOVA with Tukey's test.
