# Supplementary material for: MaMHR Contributes to Conidiation, Stress Adaptation, and Virulence in Metarhizium acridum
Source: J Fungi (Basel). 2026 Sep 24;12(10):718. doi: 10.3390/jof12100718 (PMC13706419; doi:10.3390/jof12100718)
Supplement: Supplementary file 1 [file jof-12-00718-s001.zip › Table S1.Primers used in this work..pdf]

Primers	Sequence (5'-3')	Remarks
MaMHR-LF	gacggccagtgccaagctAATATGTGTCCTGGTTTCG	Used to clone 5' end of MaMHR
MaMHR-LR	cggatccctcgagtctagTAAGGTAGGTCATTCTCCG	
MaMHR-RF	gctggccgcccatgggatCCAAGTGATGCTAGTGACC	Used to clone 3' end of MaMHR
MaMHR-RR	atgacatgattacgaattGATTTTGTATTGCGAGATA	
Pt-R	CAGCCAAGCCCAAAAAGTG	Used for screening the MaMHR-disruption transformants
Bar-F	GCTCTACACCCACCTGCT	
MaMHR-VF	ATCGGCGATGTCTTGTGCCC	disruption transformants
MaMHR-VR	AGGTGTTTGCGGATACGAGC	
MaMHR-CPF	gacggccagtgccaagct ACCACATTCTGCCCCCTTT	Used to form pK2-MaMHR -EGFP-SUR vector
MaMHR-CPR	ccttgctcaccatggatccGGTAGTTTTTGATATCGA	
CP-VF	GTTGGCAGTGAGGTAAGTTGAC	Used for screening the MaMHR -complement transformants
EGFP-VR	AAGAAGTCGTGCTGCTTCATGTG	
Gapdh-Qf	AGATGGAGGAGTTGGTGTTG	Used to analyze the expression of MaMHR by qRT-PCR
Gapdh-qR	GACTGCCCGCATTGAGAAG	
MaMHR-qF	TATCAACCTCACCAAGACAT	
MaMHR-qR	ACCACCATTATATCCATCACT	
qPksP-F	GGCCTGCAGGAAGTCATTCT	
qPksP-R	ATGAGGAAGCCCGTCAGTTG	
qSCD-F	TGGTTGCTGCTGGGATACTG	
qSCD-R	AGAGTGTTGGTGCGAGGATG	
qTHR-F	GGCATGCCCATGGAAGAGAT	
qTHR-R	TCACCCACTCGCTATCAGGA	
qAbr1-F	TCAACTACAACTCCACCGGC	
qAbr1-R	GTAGTTGGCACCATCACCGA	
qLAC-F	CGGAGCCGGTAAGTGGAAAT	
qLAC-R	GGTTTGTGACCTGGTAGCGA	
qTyr-F	TCCTCTGAACGGGCTAGTCA	
qTyr-R	ACCAGTAAGCTTGGACACGG	
qHppD-F	GATGTGGCCTTTGAGGTGGA	
qHppD-R	GACTTCTTGCCCTCCCGAAA	
qGPX-F	TCGCTGACTACAAGGGCAAG	
qGPX-R	CTGCATGATGGGGAAGCTGA	
qSOD-1-F	CATGCCTATTACCTACAGTA	
qSOD-1-R	CTTCCAGTTGATGACCTT	
qSOD-2-F	AGACAAGGACACCAAGAC	
qSOD-2-R	GGAGGCGTAGTTGAAGAT	
qSOD-4-F	GGTCAAAGCTGTTGCCGTTC	
qSOD-4-R	CGCCAAAGGTGTGAATGTGG	
qMnSOD-F	CTCCTCTTCTGAACTTCC	

qMnSOD-R	CCGAAATCCTCATTGATG
qCAT-1-F	GATACGGTAACAGCAATGG
qCAT-1-R	TCGGTCAAGTCAGATACG
qCAT-2-F	TTCTCGCCATCCAATCTC
qCAT-2-R	TCGTCGTTATTCCGTCTC
qCAT-3-F	CCTCTACTCTTCATACCTTGT
qCAT-3-R	GTCGCCATTCTCATTCAC
qCAT-4-F	AAGTTCCCGTTGTTCATC
qCAT-4-R	TTCCTGGTGAGTTGAGAG
qPOD-F	TGTCGTGTCCAACCTGTACG
qPOD-R	CGCTGTTGGCGATAATGACG
qRAD2-F	CGTTCCGACTATTGTATTACC
qRAD2-R	ACAGGCTTGAGTATCAGTAG
qRAD3-F	ATCACTCCTGACGGCCTAGT
qRAD3-F	CTTCTGAGACTTTGCCCCGT
qRAD4-F	AAATGCGAAGGAAGCAGGGA
qRAD4-R	TCGAACGAGTTGCTGCAGAT
qRAD10-F	CAGCCCGTACCCCAAAGAAT
qRAD10-R	GCAGGTTGTGAGTCCCATGA
qRAD14-F	ACATGATGCTGTTCCTGCGA
qRAD14-R	TCTGTTCGAGTCTTGCGCTT
qRAD16-F	AAACACGACTTTTGCCGAGC
qRAD16-R	ATGCGTTGTCTGAACGGAGT
qRAD23-F	AGGAAGTGGTAGTGGCAACG
qRAD23-R	GAGCTGCTGAAACTGAGGGT
qRAD25-F	CAAGAGGTCACGCACTCCTT
qRAD25-R	CCACTCTTGGCTCGACCATT
qHSP40-1-F	GGCGAAGCGGATCAATTTCC
qHSP40-1-R	CATACCTTCGCCAGCGATCT
qHSP40-2-F	CACGACCGAGTTTCAGTGGA
qHSP40-2-R	TGAGAGTGACAGTCGTTGGC
qHSP70-1-F	TGTCGATTTGGGCACCACTT
qHSP70-1-R	GTAGACAATGGCGGTTGGGA
qHSP70-2-F	GCTTGATGTTGCCCCTCTCT
qHSP70-2-R	TGACGCTCACCCTCAAAGAC
qHSP70-3-F	AGTCCTACATTGGCCGTGTG
qHSP70-3-R	CTTAAGGGCAAGCTCCTGCT
qHSP90-F	AAGAAGGTTGAGGCTGACGG
qHSP90-R	GAGCTTGTGAATGCGCTCAG
